# Dynamics of Genotypic and Phenotypic Antibiotic Resistance in a Conventional Wastewater Treatment Plant in 2 Years

**DOI:** 10.3389/fmicb.2022.898339

**Published:** 2022-08-11

**Authors:** Hanseob Shin, Yeonghyeon Kim, Shahbaz Raza, Tatsuya Unno, Song-Hee Ryu, Hor-Gil Hur

**Affiliations:** ^1^School of Earth Sciences and Environmental Engineering, Gwangju Institute of Science and Technology (GIST), Gwangju, South Korea; ^2^Faculty of Biotechnology, College of Applied Life Sciences, SARI, Jeju National University, Jeju-si, South Korea; ^3^Residual Agrochemical Assessment Division, National Institute of Agricultural Sciences, Wanju-gun, South Korea

**Keywords:** antibiotic resistance, antibiotic resistance gene (ARG), antibiotic resistant bacteria (ARB), wastewater treatment plant (WWTP), multidrug resistance, high-throughput real time PCR

## Abstract

Wastewater treatment plants (WWTPs) are considered a sink and a source of antibiotic resistance. In this study, we applied both culture-dependent and SmartChip-based culture-independent approaches for the investigation of antibiotic-resistant bacteria (ARB) and antibiotic resistance genes (ARGs) at Jungnang (JN), located in the Han River, Seoul, South Korea, for 2 years, i.e., 2017 and 2018. The JN WWTP reduced the diversity and abundance of ARB and ARGs but was not sufficient for removing them all. Interestingly, through the treatment process in the JN WWTP, the composition of diverse multidrug-resistant (MDR) bacteria was concentrated mainly into some genera of the Gammaproteobacteria class (*Citrobacter*, *Escherichia-Shigella*, and *Stenotrophomonas*), which could be key carriages to spread ARGs into the environments. In addition, SmartChip analyses showed that the relative abundance and the number of ARGs were significantly decreased from the influents to the effluents in both 2017 and 2018. SmartChip analyses for 2 years also allowed to notify the core ARGs in the influents and the effluents with the presence of clinically relevant core ARGs, such as *vanC*, *bla*_*OXA*_, and *bla*_*NDM*_, which persisted in the treatment process. Considering diverse bacterial mechanisms for exchanging and transferring ARGs, the occurrence of MDR bacteria and core ARGs could be a source for the blooming of the antibiotic resistome in the WWTP and nearby environments.

## Introduction

There is an emerging global concern related to wastewater treatment plants (WWTPs), which are considered reservoirs of antibiotic-resistant bacteria (ARB) and antibiotic-resistance genes (ARGs) (Gatica and Cytryn, 2013; Laht et al., 2014; Mao et al., 2015; Di et al., 2016; Tang et al., 2016). Due to the inflow of wastewater from various environmental sources, such as hospitals, households, or farms, ARB can share ARGs *via* horizontal gene transfer (HGT) between bacterial strains. It is known that contaminants such as toxic metals or antibiotics in the mixed influent of WWTPs may promote antibiotic resistance in ARB by means of selective pressure among them (Marti et al., 2013; Xu et al., 2015). According to a previous study, subinhibitory levels of antibiotics are known to trigger the HGT of ARGs (Carey and McNamara, 2015; Carey et al., 2016).

Some studies have suggested that even though the reduction of the abundance of ARGs was observed, a large amount of ARGs still remained in the final effluent of WWTPs. A study reported that a number of resistance genes to tetracycline and sulfonamide were present in the ARB isolated from the final effluent of a WWTP in Michigan (Munir et al., 2010; Gao et al., 2012). Another study showed 140 clinically important ARGs conferring resistance to nine classes of antibiotics to be present in plasmids in bacteria obtained from the effluent of a WWTP in Germany (Szczepanowski et al., 2009). A study examining the treatment process of WWTPs showed the level of antibiotic resistance of *Acinetobacter* spp. to be higher in the effluent than in the influent (Zhang et al., 2009). However, other studies have shown a relative decrease in the abundance of ARGs in the effluent compared with that in the influent, suggesting that the treatment process of WWTPs is not to be considered a source of ARG contamination (Karkman et al., 2016). A metagenomic analysis demonstrated the efficient reduction of ARGs in a WWTP with the reduction of potentially pathogenic bacteria (Tang et al., 2016). Statistically significant reductions in levels of *tet (A)*, *tet (L)*, *tet (O)*, *tet (W)*, and *tet (X)* genes have been reported (LaPara, 2010).

Therefore, it was necessary to investigate the distribution of ARB and ARGs that may impact the *in situ* fate of antibiotic resistance in aquatic environments in South Korea. We performed experiments for 2 years, i.e., 2017 and 2018, at the Jungnang (JN) WWTP, using culture-dependent and SmartChip-based culture-independent approaches. The treatment process in the JN WWTP was seen to reduce the abundance of ARB and ARGs but not eliminate them. We also observed that multidrug-resistant (MDR) bacterial diversity converged into mainly one class of Gram-negative Gammaproteobacteria class, with *Citrobacter*, *Escherichia-Shigella*, and *Stenotrophomonas* being the most commonly found MDR bacteria. In addition, the SmartChip-based study confirmed the vast amount of ARGs present in the influent and effluent and indicated the presence of clinically relevant ARGs, such as *vanC*, *bla*_*OXA*_, and *bla*_*NDM*_ in the effluent.

## Materials and Methods

### Collection and Processing of Samples

**FIGURE 1:**
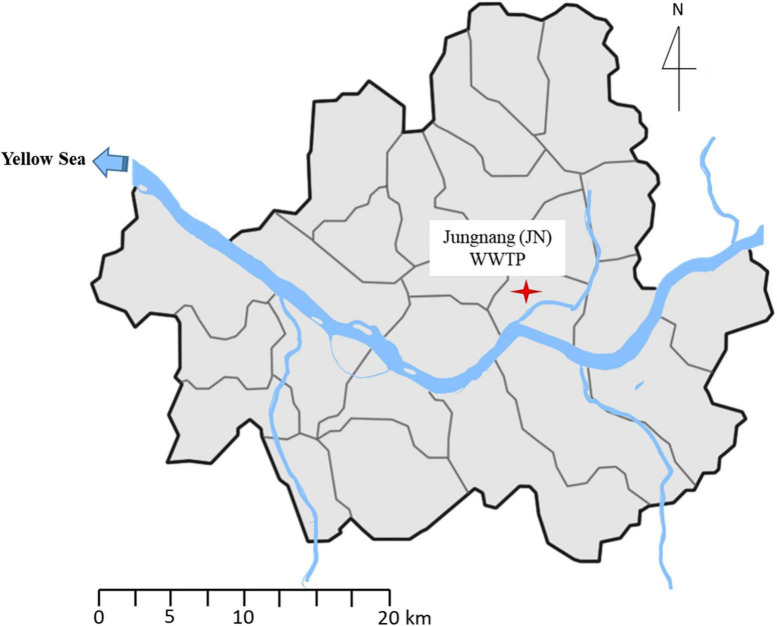
Location of Jungnang JN Wastewater treatment plant (WWTP) on the Han River, Seoul, South Korea.

Influent (4 L) and effluent samples (20 L) were collected in duplicate in sterile bottles from JN WWTP (N 127.062884 E 37.5598383) once a year (in May) on the Han River, Seoul, South Korea (Figure 1) in May 2017 and 2018. The samples were immediately transferred to the laboratory under cool condition (4%). The duplicate samples were mixed, and 1 L of the influent and effluent samples were filtered through a 0.22 μm pore size membrane filter (Advantec, Tokyo, Japan) for isolation of bacteria. Then, the membranes were placed in 10 ml of Mueller-Hinton (MH) broth (MBCell, Seoul, South Korea), vortexed thoroughly, and serially diluted up to 10^–3^ times. Suspensions (100 μl) were spread on MH agar plates, and the plates were incubated at 37% for 48 h. After incubation, we selected the colonies regardless of their size, color, and shape, and the colonies were streaked on new MH agar plates to obtain a single colony. The isolates were then stored in a freezer at −80°C for culture-based experiments. Notably, 1 and 10 L of the influent and effluent samples were also filtered through a 0.22 μm pore size membrane filter (Advantec, Tokyo, Japan) to extract environmental DNA for SmartChip Real-time PCR. DNA extraction was performed using a PowerWater DNA isolation kit (MoBio Laboratories Inc., CA, United States) according to the manufacturer’s instructions. The extracted DNA was stored in a freezer at -80% for further SmartChip Real-Time PCR.

### Phenotypic and Genotypic Resistance Tests

An antibiotic susceptibility test was performed using the agar dilution method (Wiegand et al., 2008) using all isolates obtained. *Escherichia coli* ATCC25922 was used as the quality control. Five antibiotics belonging to the following four antibiotic classes were used for the resistance tests: aminoglycoside (16 mg/L gentamycin; Gen), β-lactam (8 mg/L amoxicillin; Amx and 8 mg/L meropenem; Mer), sulfonamide (32 mg/L sulfamethoxazole; Sul), and tetracycline (16 mg/L tetracycline; Tet). Those antibiotics are known to belong to the groups of antibiotics widely used for therapeutic purposes. All isolates were streaked on MH agar plates containing each antibiotic individually and incubated at 28% for 24 h. The colonies grown on the plates were determined to be ARB. The isolates showing resistance to four or five antibiotics were taxonomically identified by sequencing of 16S rDNA (Macrogen, Seoul, South Korea) referring to the database of the National Center for Biotechnology Information (NCBI) and subjected to amplification of 14 ARGs belonging to the following four classes: aminoglycoside [*strA*, *strB*, *aph (3′)-IIIa*, *aph (2″)-Ib*, *aac (6′)-Ie-aph (2″)-Ia*, and *ant (4′)-Ia*], sulfonamide (*sul1* and *sul2*), tetracycline (*tetA*, *tetB*, and *tetM*), and β-lactam (*bla*_*TEM*_, *bla*_*CTX*–*M*–1_, and *bla*_*OXA*–1_) as described in previous studies (Vakulenko et al., 2003; Ogutu et al., 2015; Pyatov et al., 2017).

### High-Throughput Quantitative Real-Time PCR

Quantitative PCR was performed using a SmartChip Real-time PCR system (WaferGen Inc., California state, United States), targeting 343 ARGs (Zhu et al., 2013). The 343 ARGs in this study conferred resistance to almost all classes of antibiotics. Quantitative amplification was performed with a 100 nl volume containing 1 × LightCycler 480 SYBR^®^ Green I Master Mix (Roche Inc., Basel, Switzerland), approximately 5 ng/μl DNA template, 1 μM of each forward and reverse primers, and nuclease-free PCR-grade water. The reaction was performed under the following conditions: initial denaturation at 95% for 10 min, followed by 40 cycles of denaturation at 95% for 30 s, annealing at 60% for 30 s, and a melting curve analysis auto-generated by the program. Amplification was conducted in triplicates, and the housekeeping gene such as *rpoB*, *mdh*, and *gapA* was amplified as a positive control. The results were analyzed using the SmartChip qPCR software (version 2.7.0.1). Amplification efficiency ranging between 1.8 and 2.2 was used for PCR, and a threshold cycle (Ct) of 31 was used as the detection limit in accordance with a previous study (Zhu et al., 2013). Three replicates of samples were analyzed. For the analysis of the relative abundance of ARGs, the copy number of ARGs was calculated using an equation described in a previous study (Looft et al., 2012) and normalized using the copy number of 16S rDNA. Primer sequences and PCR efficiencies are provided in Supplementary Tables 1, 2. In addition, the significance test (Wilcox test) was performed using R studio version 1.1.463 after the normality test (Shapiro test).

## Results

### Phenotypic Resistance Profile of the Bacterial Community

A total of 960 bacterial isolates (240 isolates from each sample) were obtained from the influent and effluent of the JN WWTP in 2017 and 2018. In 2017, 38 and 58 MDR bacterial isolates, showing resistance to four or five antibiotics (Amx, Gen, Mer, Sul, and Tet), were obtained in the influent and effluent, respectively. In 2018, 44 and 36 MDR bacterial isolates were obtained from the influent and effluent, respectively. Among the seven classes observed, namely, Actinobacteria, Bacteroidia, Betaproteobacteria, Epsilonproteobacteria, Flavobacteriia, Gammaproteobacteria, and Sphingobacteriia, Gammaproteobacteria and Flavobacteriia were found as the most dominant classes present in samples of both, influent and effluent, obtained in 2017 and 2018 (Tables 1, 2). The ratio (%) of the number of Gammaproteobacteria to the total number of different types of bacteria decreased from influent (77–84%) to effluent (53–56%) during the study period. At the genus level, 12 bacterial genera were observed from influent in 2017 and in 2018, while only 10 and 11 genera were isolated from the effluent in 2017 and 2018, respectively (Tables 1, 2). In the 2017 influent, *Acinetobacter* (*n* = 15) was the most dominant genus, followed by *Escherichia* (*n* = 8), *Escherichia-Shigella* (*n* = 3), and *Pseudomonas* (*n* = 3). However, in the effluent of 2017, *Stenotrophomonas* (*n* = 22) comprised the majority of the MDR bacteria, followed by *Chryseobacterium* (*n* = 11), *Acinetobacter* (*n* = 8), and *Microbacterium* (*n* = 8). In the influent of 2018, *Acinetobacter* (*n* = 13) was also the most dominant, followed by *Chryseobacterium* (*n* = 7), *Pseudomonas* (*n* = 7), and *Escherichia-Shigella* (*n* = 4). In the effluent of 2018, *Chryseobacterium* (*n* = 8) was the most dominant, followed by *Acinetobacter* (*n* = 7), *Pseudomonas* (*n* = 6), *Citrobacter* (*n* = 4), *Elizabethkingia* (*n* = 4), and *Sphingobacterium* (*n* = 4). Based on the phenotypic analyses of antibiotic resistance in this study, we found four types of antibiotic resistance patterns, including Amx-Gen-Mer-Sul-Tet, Amx-Gen-Mer-Tet, Amx-Gen-Sul-Tet, and Amx-Mer-Sul-Tet from both, the influent and the effluent in 2017 and 2018 (Tables 1, 2). It should be noted that the number of *Acinetobacter* decreased from the influent to the effluent for 2 years, while that of *Chryseobacterium* increased. Interestingly, most of the genera, namely, *Acinetobacter, Escherichia, Escherichia-Shigella*, and *Stenotrophomonas*, belonging to the class Gammaproteobacteria, seemed to be the main carriers of ARGs at the influents. In particular, at the effluents, *Citrobacter*, *Escherichia-shigella*, *Stenotrophomonas*, and *Providencia* were the carriers of ARGs in 2017 and 2018. The diversity of ARG-carrying genera was decreased from the influents to the effluents in both years.

**TABLE 1:** Phenotypic and genotypic resistance profile of multidrug-resistant bacteria isolated from Jungnang (JN) wastewater treatment plant (WWTP) in 2017.

Class	Genus	Phenotypic resistance	Genotypic resistance*														No. of isolates
			Amx, Mer			Gen						Sul		Tet			
			a*	b*	c*	d*	e*	f*	g*	h*	i*	j*	k*	l*	m*	n*	
***Isolated from influent, 2017***																	

Betaproteobacteria	*Comamonas*	Amx-Gen-Sul-Tet	**√**		**√**	**√**	**√**					**√**	**√**				1
	*Neisseria*	Amx-Mer-Sul-Tet															1
	*Vitreoscilla*	Amx-Gen-Mer-Sul-Tet	**√**			**√**	**√**						**√**	**√**			1
Epsilonproteobacteria	*Arcobacter*	Amx-Mer-Sul-Tet															2
Flavobacteria	*Chryseobacterium*	Amx-Gen-Mer-Sul-Tet	**√**			**√**	**√**						**√**	**√**			1
Gammaproteobacteria	*Acinetobacter*	Amx-Gen-Mer-Sul-Tet	**√**			**√**	**√**						**√**	**√**			4
			**√**			**√**	**√**					**√**	**√**	**√**			1
			**√**			**√**	**√**						**√**				3
		Amx-Gen-Sul-Tet	**√**			**√**	**√**						**√**	**√**			1
			**√**			**√**	**√**						**√**				1
			**√**			**√**	**√**		**√**				**√**	**√**			1
		Amx-Mer-Sul-Tet															1
			**√**			**√**	**√**						**√**				1
																	2
	*Escherichia*	Amx-Gen-Mer-Sul-Tet	**√**			**√**	**√**						**√**	**√**			2
						**√**	**√**						**√**	**√**			1
								**√**								**√**	1
		Amx-Gen-Sul-Tet		**√**	**√**												2
			**√**			**√**	**√**						**√**	**√**			1
			**√**			**√**	**√**						**√**				1
	*Escherichia-Shigella*	Amx-Gen-Mer-Sul-Tet	**√**			**√**	**√**						**√**	**√**			3
	*Pseudomonas*	Amx-Mer-Sul-Tet															3
	*Shigella*	Amx-Gen-Sul-Tet											**√**	**√**			1
	*Stenotrophomonas*	Amx-Gen-Mer-Sul-Tet															1
	*Yokenella*	Amx-Gen-Sul-Tet	**√**			**√**	**√**						**√**				1
Actinobacteria	*Microbacterium*	Amx-Gen-Mer-Sul-Tet															1
		Amx-Mer-Sul-Tet															7
	*Rhodococcus*	Amx-Mer-Sul-Tet															1
Flavobacteriia	*Chryseobacterium*	Amx-Gen-Mer-Sul-Tet															8
		Amx-Gen-Mer-Sul															1
		Amx-Mer-Sul-Tet															2
	*Empedobacter*	Amx-Gen-Mer-Sul															1
Gammaproteobacteria	*Acinetobacter*	Amx-Gen-Mer-Sul-Tet															1
		Amx-Mer-Sul-Tet															7
	*Escherichia-Shigella*	Amx-Mer-Sul-Tet	**√**	**√**		**√**	**√**		**√**				**√**	**√**			1
	*Stenotrophomonas*	Amx-Gen-Mer-Sul-Tet															17
		Amx-Mer-Sul-Tet	**√**			**√**	**√**					**√**	**√**	**√**			1
																	4
	*Sphingobacterium*	Amx-Gen-Mer-Sul-Tet					**√**										1
Sphingobacteria																	4
		Amx-Mer-Sul-Tet															1

*a: bla_TEM_, b: bla_CTX–M–1_, c: bla_OXA–1_, d: strA, e: strB, f: aph (3′)-IIIa, g: aph (2″)-Ib, h: aac (6′)-Ie-aph (2″)-Ia, i: ant (4′)-Ia, j: sul1, k: sul2, l: tetA, m: tetB, n: tetM.

**TABLE 2:** Phenotypic and genotypic resistance profile of multidrug-resistant bacteria isolated from JN WWTP in 2018.

Class	Genus	Phenotypic resistance	Genotypic resistance*														No. of isolates
			Amx, Mer			Gen						Sul		Tet			
			a*	b*	c*	d*	e*	f*	g*	h*	i*	j*	k*	l*	m*	n*	
***Isolated from influent, 2018***																	

Bacteroidia	*Elizabethkingia*	Amx-Mer-Sul-Tet															1
Flavobacteriia	*Chryseobacterium*	Amx-Gen-Mer-Sul-Tet															3
						**√**	**√**						**√**				1
													**√**				1
												**√**	**√**				1
		Amx-Mer-Sul-Tet															1
Gammaproteobacteria	*Acinetobacter*	Amx-Gen-Mer-Sul-Tet															1
		Amx-Gen-Sul-Tet				**√**	**√**										2
																	1
			**√**														1
		Amx-Mer-Sul-Tet															8
	*Aeromonas*	Amx-Gen-Mer-Sul-Tet										**√**	**√**				1
	*Citrobacter*	Amx-Gen-Mer-Sul-Tet	**√**														1
		Amx-Gen-Sul-Tet	**√**														2
	*Escherichia-Shigella*	Amx-Gen-Mer-Sul-Tet															1
		Amx-Gen-Sul-Tet	**√**	**√**	**√**	**√**	**√**										1
			**√**														1
		Amx-Mer-Sul-Tet															1
	*Proteus*	Amx-Gen-Sul-Tet															1
		Amx-Mer-Sul-Tet															2
	*Pseudomonas*	Amx-Gen-Mer-Sul-Tet															1
		Amx-Gen-Sul-Tet	**√**	**√**		**√**	**√**										1
		Amx-Mer-Sul-Tet										**√**	**√**				1
													**√**				2
																	2
	*Raoultella*	Amx-Gen-Mer-Sul-Tet															1
	*Stenotrophomonas*	Amx-Gen-Mer-Sul-Tet															1
	*Uruburuella*	Amx-Gen-Sul-Tet			**√**												1
Sphingobacteriia	*Sphingobacterium*	Amx-Gen-Sul-Tet															2
***Isolated from effluent, 2018***																	
Betaproteobacteria	*Burkholderia*	Amx-Gen-Sul-Tet															1
Bacteroidia	*Elizabethkingia*	Amx-Gen-Mer-Sul-Tet															3
		Amx-Gen-Sul-Tet															1
Flavobacteriia	*Chryseobacterium*	Amx-Gen-Mer-Sul-Tet															2
		Amx-Gen-Sul-Tet															6
Gammaproteobacteria	*Acinetobacter*	Amx-Gen-Mer-Sul															2
		Amx-Gen-Mer-Sul-Tet															1
		Amx-Gen-Sul-Tet															2
		Amx-Mer-Sul-Tet															2
	*Aeromonas*	Amx-Gen-Sul-Tet															1
	*Citrobacter*	Amx-Gen-Sul-Tet	**√**									**√**	**√**				1
			**√**														1
			**√**			**√**								**√**	**√**		1
																	1
	*Escherichia-Shigella*	Amx-Gen-Sul-Tet	**√**										**√**				2
	*Stenotrophomonas*	Amx-Gen-Sul-Tet				**√**	**√**					**√**					1
	*Pseudomonas*	Amx-Gen-Mer-Sul-Tet	**√**														4
		Amx-Gen-Mer-Sul															2
	*Providencia*	Amx-Gen-Sul-Tet				**√**											1
Sphingobacteriia	*Sphingobacterium*	Amx-Gen-Sul-Tet															1
		Amx-Gen-Mer-Sul-Tet															3

*a: bla_TEM_, b: bla_CTX–M–1_, c: bla_OXA–1_, d: strA, e: strB, f: aph (3′)-IIIa, g: aph (2″)-Ib, h: aac (6′)-Ie-aph (2″)-Ia, i: ant (4′)-Ia, j: sul1, k: sul2, l: tetA, m: tetB, n: tetM.

Among the phenotypic patterns of antibiotic resistance, the set Amx-Gen-Mer-Sul-Tet was dominant having *n* = 18 (47.4%) and *n* = 32 (55.2%) in the influent and effluent of 2017, respectively. The next dominant set of antibiotic resistance, Amx-Mer-Sul-Tet, in the influent and effluent of 2017 had *n* = 10 (26.3%) and *n* = 24 (41.3%), respectively. Thus, the antibiotic resistance patterns of Amx-Gen-Mer-Sul-Tet and Amx-Mer-Sul-Tet of MDR bacteria were dominant at *n* = 18 and *n* = 10 in the influent, respectively and at *n* = 32 and *n* = 23 in the effluent of 2017, respectively. In addition, the dominant antibiotic resistance set of Amx-Gen-Mer-Sul-Tet was found in the majority of ARB, namely, *Acinetobacter* (*n* = 8), *Escherichia* (*n* = 4), and *Escherichia-Shigella* (*n* = 3) in the influent of 2017 and *Stenotrophomonas* (*n* = 17), *Chryseobacterium* (*n* = 8), *Sphingobacterium* (*n* = 5), and *Acinetobacter* (*n* = 1) in the effluent. The second most dominant antibiotic resistance set of Amx-Mer-Sul-Tet was found in the majority of ARB, namely, *Acinetobacter* (*n* = 4) and *Pseudomonas* (*n* = 3) in the influent of 2017 and *Acinetobacter* (*n* = 7), *Microbacterium* (*n* = 7), *Stenotrophomonas* (*n* = 5), *Chryseobacterium* (*n* = 2), *Escherichia-Shigella* (*n* = 1), *Rhocococcus* (*n* = 1), and *Sphingobacterium* (*n* = 1) in the effluent. In the influent and effluent of 2018, the sets Amx-Mer-Sul-Tet and Amx-Gen-Sul-Tet were dominant, with *n* = 18 (40.1%) and *n* = 20 (55.6%), respectively. The third most dominant set of antibiotic resistance, Amx-Gen-Mer-Sul-Tet, in the influent and effluent of 2018 was *n* = 13 (29.5%) and *n* = 13 (36.1%), respectively. In addition, the Amx-Mer-Sul-Tet set was also found to be the second most dominant in the influent of 2018, with *n* = 13 (29.5%). Thus, the sets Amx-Gen-Mer-Sul-Tet, Amx-Gen-Sul-Tet, and Amx-Mer-Sul-Tet in the influent of 2018 encompassed approximately 99.1% of the four sets of antibiotic resistance patterns, while the two sets, Amx-Gen-Mer-Sul-Tet and Amx-Gen-Sul-Tet, occupied approximately 91.6% of antibiotic resistance patterns. The dominant antibiotic resistance set of Amx-Mer-Sul-Tet was frequently found in *Acinetobacter* (*n* = 8), *Pseudomonas* (*n* = 4), and *Proteus* (*n* = 2) in the influent of 2018, and the Amx-Gen-Sul-Tet set was frequently observed in *Chryseobacterium* (*n* = 6), *Citrobacter* (*n* = 4), *Acinetobacter* (*n* = 2), and *Escherichia-Shigella* (*n* = 2) in the effluent of 2018. The second most dominant antibiotic resistance set, Amx-Gen-Mer-Sul-Tet, was in the majority of *Chryseobacterium* (*n* = 6) in the influent of 2018 and in *Pseudomonas* (*n* = 4), *Elizabethkingia* (*n* = 3), and *Sphingobacterium* (*n* = 3) in the effluent of 2018. Specifically, co-occurrence patterns of ARGs were also observed in multidrug-resistant bacteria. The genes, *bla*_*TEM*_, *strA*, *strB*, *sul2*, and *tetA*, were detected mostly together in the influent (*n* = 15) and effluent (*n* = 2) of 2017. In 2018, two co-occurrence patterns were found as *strA* and *strB* (*n* = 4 and 1) and *sul1* and *sul2* (*n* = 3 and 1) in the influent and effluent, respectively.

### Genotypic Antibiotic Resistance Profiling of Multidrug-Resistant Bacteria

All 14 ARGs were detected from MDR bacteria, and the profiles of ARGs possessed by MDR bacteria were arranged as shown in Tables 1, 2. Gammaproteobacteria class seemed to carry most of the ARGs at all sampling sites. In the Gammaproteobacteria class, *Acinetobacter* carried up to six ARGs in the influent. However, none of the ARGs were detected from *Acinetobacter* in the effluent. Some genera, *Citrobacter, Escherichia, Escherichia-Shigella, Shigella, Proteus, and Yokenella* belonging to the Enterobacteriaceae family, also possessed up to five and seven ARGs in the influent and effluent, respectively, despite a lower number of Enterobacteriaceae in the effluent. *Pseudomonas* and *Aeromonas* were also found to carry up to four and two ARGs, respectively. Specifically, co-occurrence patterns of ARGs were also observed. The genes, *bla*_*TEM*_, *strA*, *strB*, *sul2*, and *tetA*, were detected mostly together in the influent (*n* = 15) and effluent (*n* = 2) of 2017. In 2018, two co-occurrence patterns were found as *strA* and *strB* (*n* = 4 and 1) and *sul1* and *sul2* (*n* = 3 and 1) in the influent and effluent, respectively.

Among the combined genotypic patterns of antibiotic resistance found in 2017, *bla*_*TEM*_-*strA-strB-sul2-tetA* was the most dominant, having *n* = 13 (34.2%) in the influent, and was found in *Acinetobacter* (*n* = 5), *Escherichia* (*n* = 3), and *Escherichia-Shigella* (*n* = 3), *Chryseobacterium* (*n* = 1), and *Vitreoscilla* (*n* = 1). The second most dominant genotypic resistance combination in the 2017 influent was *bla*_*TEM*_*-strA-strB-sul2*, which was present in *Acinetobacter* (*n* = 5), *Escherichia* (*n* = 1), and *Yokenella* (*n* = 1). The other five genera of MDR bacteria, *Acinetobacter* (*n* = 8), *Chryseobacterium* (*n* = 11), *Empedobacter* (*n* = 1), *Microbacterium* (*n* = 8), and *Rhodococcus* (*n* = 1), which were phenotypically resistant to antibiotics, did not carry ARGs in the effluent of 2017. Although diverse genotypic patterns of antibiotic resistance were found in the influent of 2017, there were only three different types of antibiotic-resistant genotypic combinations, *strB*, *bla*_*TEM*_*-bla*_*CTX*–*M*–1_*-strA-strB-aph (2″)-Ib-sul2-tetA*, and *bla*_*TEM*_-*strA-strB-sul1-sul2-tetA* in the isolates of *Sphingobacterium* (*n* = 1), *Escherichia-Shigella* (*n* = 1), and *Stenotrophomonas* (*n* = 1), respectively, in the effluent of 2017.

The same patterns of antibiotic resistant genetic combinations in different MDR bacteria observed in the samples of 2017 were not found in the influent or effluent in the samples of 2018. Among multiple sets of antibiotic-resistant genetic combinations in the 2018 influent, only seven sets were observed. The observed sets are as follows: *strA-strB-sul2* and *sul1-sul2* in *Chryseobacterium* (*n* = 2), *strA-strB* in *Acinetobacter* (*n* = 2), *sul1-sul2* in *Aeromonas* (*n* = 1), *bla*_*TEM*_*-bla*_*CTX*–*M*–1_-*bla*_*OXA*–1_-*strA-strB* in *Escherichia-Shigella* (*n* = 1), *bla*_*TEM*_*-bla*_*CTX*–*M*–1_-*strA-strB*, and *sul1* and *sul2* in *Pseudomonas* (*n* = 2). Table 3 shows that, in general, the total number of ARGs detected from MDR bacteria decreased from influent (*n* = 27–83) to effluent (*n* = 14). ARGs present in abundance (*n* = 126), such as *sul2* (*n* = 27), *bla*_*TEM*_ (*n* = 28), *strA* (*n* = 25), *strB* (*n* = 24), *tetA* (*n* = 14), and *sul1* (*n* = 8) comprised more than 91% of the 138 ARGs counted. In contrast, the genes *aph (3’)-IIIa, aph (2″)-Ib, aac (6′)-Ie-aph (2″)-Ia*, *ant (4′)-Ia*, *tetB*, and *tetM* were rarely found (*n* = 0–2).

**TABLE 3:** Detection of antibiotic resistance genes (ARGs) from multidrug-resistant bacteria at the influent and effluent in 2017 and 2018.

Class	ARGs	2017		2018		Total
		Influent	Effluent	Influent	Effluent	
Aminoglycoside	*strA*	16	2	4	3	25
	*strB*	16	3	4	1	24
	*Aph (3′)-IIIa*	1	0	0	0	1
	*Aph (2″)-Ib*	1	1	0	0	2
	*Aac (6′)-Ie-aph (2″)-Ia*	0	0	0	0	0
	*Ant (4′)-Ia*	0	0	0	0	0
β-Lactam	*bla*_*TEM*_	15	2	6	5	28
	*bla*_*CTX*–*M*–1_	1	1	2	0	4
	*bla*_*OXA*–1_	2	0	2	0	4
Sulfonamide	*sul1*	2	1	3	2	8
	*sul2*	17	2	6	2	27
Tetracycline	*tetA*	11	2	0	1	14
	*tetB*	0	0	0	0	0
	*tetM*	1	0	0	0	1
**Total**		**83**	**14**	**27**	**14**	**138**

### Relative Abundance of Antibiotic Resistance Genes Measured Using SmartChip Analyses

**FIGURE 2:**
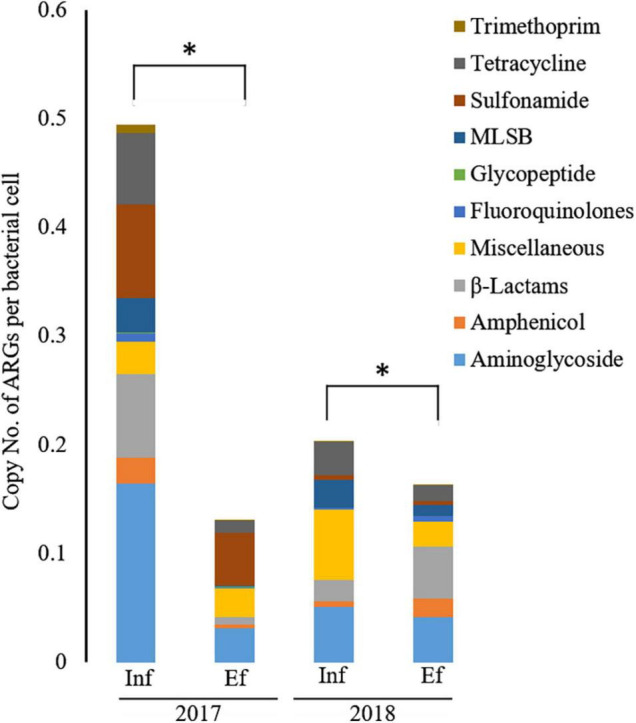
The relative abundance of antibiotic resistance genes (ARGs) from the influent (Inf) and the effluent (Ef) of wastewater treatment plants through SmartChip analyses. The copy number of ARGs was normalized by that of 16S rDNA, and it represented the relative abundance of ARGs. Each color indicates the classes of ARGs. An asterisk (*) denotes a statistically significant difference (Wilcox test; *p* < 0.05) between influents and effluents of the WWTP.

Overall, the relative abundance of ARGs in terms of the copy number per bacterial cell was significantly decreased (*p* < 0.05) from the influent (0.20–0.50) to effluent (0.13–0.16) in 2017 and 2018 (Figure 2). However, in 2017, resistance genes to miscellaneous and glycopeptide classes were more abundant in the effluent (0.047 and 0.003 copy number per bacterial cell, respectively) than in the influent (0.027 and 0.0008 copy number per bacterial cell, respectively). In 2018, genes conferring resistance to amphenicol, β-lactam, fluoroquinolone, and trimethoprim were higher in the effluent (0.017, 0.048, 0.005, and 0.001 copy number per bacterial cell, respectively) than in the influent (0.005, 0.020, 0.002, and 0.0003 copy number per bacterial cell, respectively). In the classes of ARGs, entirely at both influent and effluent in 2017 and 2018, the abundance of aminoglycoside resistance genes were most abundant, in terms of copy number per bacterial cell, as 0.286, followed by tetracycline as 0.163, miscellaneous genes as 0.144, sulfonamide as 0.116, MLSB as 0.110, β-lactam as 0.090, and other minor classes (amphenicol, glycopeptide, fluoroquinolone, and trimethoprim). In the influent of 2017, aminoglycoside resistance genes were most abundant in terms of copy number per bacterial cell and the most dominant having a copy number of 0.164 (33.2%), followed by sulfonamide (copy number = 0.862, 17.4%), β-lactam (copy number = 0.077, 15.6%), and tetracycline (copy number = 0.066, 13.4%) while the least dominant class was glycopeptide as copy number of 0.0008 (0.2%). In the effluent of 2017, sulfonamide resistance genes were most dominant having copy number of 0.049 (37.3%), followed by classes of aminoglycoside (copy number = 0.031, 24.1%), miscellaneous genes (copy number = 0.026, 20.4%), tetracycline (copy number = 0.011, 8.1%), and β-lactam (copy number = 0.007, 5.3%) while the least dominant class was trimethoprim (copy number = 0.00013, 0.1%). In the influent of 2018, class of miscellaneous genes was most dominant having copy number of 0.064 (31.8%), followed by aminoglycoside (copy number = 0.051, 25.1%), tetracycline (copy number = 0.030, 15.0%), MLSB (copy number = 0.026, 12.6%), β-lactam (copy number = 0.020, 9.7%), and amphenicol (copy number = 0.005, 2.5%), while the least dominant class was trimethoprim (copy number = 0.0003, 0.15%). In the effluent of 2018, β-lactam resistance genes were most dominant having copy number of 0.048 (29.4%), followed by aminoglycoside (copy number = 0.041, 25.2%), miscellaneous genes (copy number = 0.023, 14.2%), amphenicol (copy number = 0.071, 10.6%), and tetracycline (copy number = 0.014, 8.5%), while the least dominant class was glycopeptide (copy number = 0.00015, 0.09%). Taken together, the most abundant ARGs in the influent and effluent of the JN WWTP are in the category of antibiotic resistance to aminoglycoside, β-lactam, sulfonamide, and tetracycline.

### Occurrence Patterns of Antibiotic Resistance Genes

**FIGURE 3:**
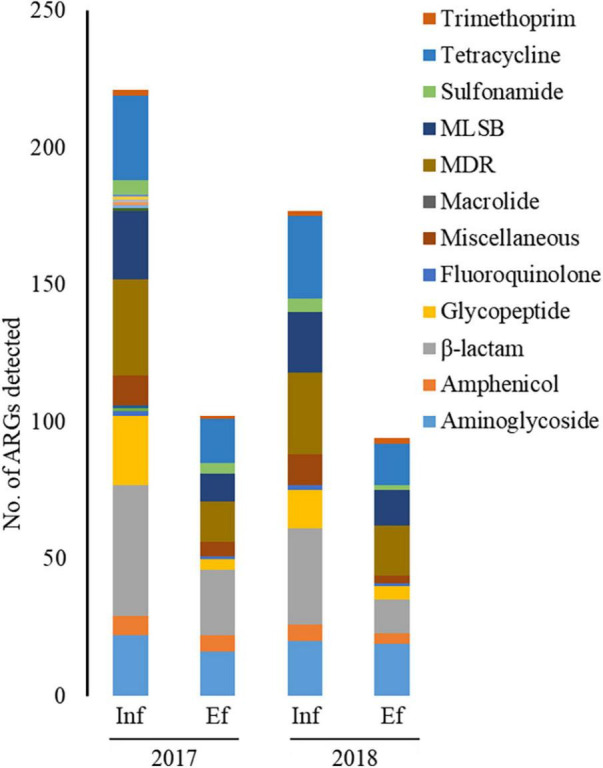
The number of ARGs from the influent (Inf) and the effluent (Ef) of wastewater treatment plants through SmartChip analyses. The number of detected ARGs was shown by ARG classes in the influent and effluent of 2017 and 2018. Each color indicates the classes of ARGs.

**FIGURE 4:**
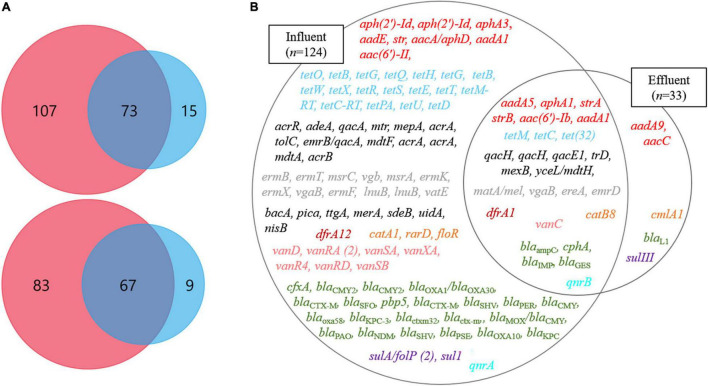
The ARGs at influents (Inf) and effluents (Ef) of wastewater treatment plants through SmartChip analyses. **(A)** The Venn diagrams represent the shared ARGs between influents (pink) and effluents (blue) in 2017 (top) and 2018 (bottom). **(B)** The Venn diagram on the right side represents the core ARGs between influents (left circle) and effluents (right circle). Each color indicates a class of ARGs as red (aminoglycoside), sky-blue (tetracycline), gray (macrolide), black (miscellaneous), brown (trimethoprim), orange (Amphenicol), green (β-lactam), purple (sulfonamide), pink (glycopeptide), and mint (fluoroquinolone).

Through the SmartChip analyses, a total of 224 ARGs were detected in the influent and effluent of the WWTP in 2017 and 2018, which belong to eleven classes conferring resistance to aminoglycosides, amphenicol, β-lactams, fluoroquinolones, glycopeptides, macrolide-lincosamide-streptogramin B (MLSB), MDR, sulfonamide, tetracycline, trimethoprim, and miscellaneous. Figure 3 shows that the total number of ARGs detected was reduced from *n* = 180 in 2017 and *n* = 150 in 2018, as in the influent, to *n* = 88 in 2017 and *n* = 76 in 2018, as in the effluent. Notably, 77.4% of ARGs (96/124, the number of ARGs) were reduced at the effluent of the JN WWTP, while 22.6% of ARGs persisted. The number of ARGs to β-lactam, which were the majority of ARGs in the influent of 2017 and 2018, was found to be reduced from the influent (*n* = 48 in 2017 and *n* = 35 in 2018) to the effluent (*n* = 24 in 2017 and *n* = 12 in 2018). The number of ARGs from the influent to the effluent in both years also showed similar patterns of reduction in the resistance genes to all antibiotics except for trimethoprim. Despite a reduction in the number of ARGs from the influent to the effluent, high numbers of co-occurring ARGs between the influent and effluent were observed as *n* = 73 and *n* = 67 in 2017 and 2018, respectively (Figure 4A). This co-occurrence of ARGs could indicate the origin as well as the recirculation of the ARGs in the WWTP. In addition, 15 and nine ARGs, which were not detected in the influent, were observed in the effluent of 2017 and 2018, respectively.

We defined the ARGs that were detected in both years, 2017 and 2018, as the core ARGs (Figure 4B) that could facilitate the understanding of the presence of persistent ARGs in the influent and effluent of WWTPs. Among the total core ARGs (*n* = 129), the core ARGs found in each influent and effluent were 124 and 33, respectively. The influent and the effluent had 28 shared ARGs, which were considered as the core ARGs of the JN WWTP. The ARGs in the influent comprised resistant genes to aminoglycoside (*n* = 8), amphenicol (*n* = 3), β-lactam (*n* = 23), fluoroquinolone (*n* = 1), glycopeptide (*n* = 7), MDR (*n* = 13), MLSB (*n* = 12), sulfonamide (*n* = 2), trimethoprim (*n* = 1), tetracycline (*n* = 18), and miscellaneous antibiotics (*n* = 7) while those in the effluent consisted of resistance genes to aminoglycoside (*n* = 2), amphenicol (*n* = 1), β-lactam (*n* = 1), and sulfonamide (*n* = 1). The core ARGs that survived the treatment process performed in the JN WWTP, and later get discharged into the environment, comprised those with resistance to aminoglycoside (*n* = 6), tetracycline (*n* = 3), MDR (*n* = 7), MLSB (*n* = 4), trimethoprim (*n* = 1), amphenicol (*n* = 1), glycopeptide (*n* = 1), β-lactam (*n* = 4), and fluoroquinolone (*n* = 1). In addition, clinically relevant ARGs, such as aminoglycoside resistance genes (*aadA1*, *aphA3*, *aacC*, *strA*, *and strB*), β-lactam resistance genes (*bla*_*KPC*_, *bla*_*NDM*_, *bla*_*OXA*_, *bla*_*IMP*_), glycopeptide resistance genes (*vanC*, *vanD*), sulfonamide resistance genes (*sul1 and sulIII*), and tetracycline resistance genes (*tetB*, *tetC*, and *tetM*) were found in the influent. However, the genes *aphA3*, *bla*_*KPC*_, *bla*_*NDM*_, *vanD*, *sul1*, and *tetB* were not observed in the effluent, whereas *aacC*, *strA*, *strB*, *bla*_*IMP*_, *vanC*, *sulIII*, *tetC*, and *tetM* persisted in the effluent.

## Discussion

Wastewater treatment plants have been reported as hotspots for the proliferation of ARB and the increased level of phenotypic antibiotic resistance of bacteria (Manaia, 2006; Zhang et al., 2009; Galvin et al., 2010), suggesting that WWTPs are not effective in mitigating antibiotic resistance. However, studies reporting these results have been inconsistent (Pazda et al., 2019). In this study, performed in 2 years (2017 and 2018), the phenotypic and genotypic results showed that the treatment process used in the WWTP reduced the level of ARGs in terms of abundance and diversity effectively but was not sufficient to remove all ARGs. In fact, JN WWTP showed the presence of persistent MDR bacteria with various ARGs, and a large variety of clinically relevant ARGs remained in the effluent.

Interestingly, MDR bacteria with ARGs in the influent mostly belonged to the classes Betaproteobacteria, Flavobacteriia, and Gammaproteobacteria. Among them, *Acinetobacter, Aeromonas, Citrobacter, Escherichia, Escherichia-Shigella, Pseudomonas, Shigella, Uruburuella*, and *Yokenella*, which belonged to Gammaproteobacteria, were the major carriers of ARGs. In the effluent, MDR bacteria with ARGs were mostly concentrated in the Gammaproteobacteria class, comprising *Citrobacter, Escherichia-Shigella, Pseudomonas, Providencia*, and *Stenotrophomonas*. At this moment, it is unclear how specific genera (such as *Citrobacter, Escherichia-Shigella*, and *Stenotrophomonas*) of the Gammaproteobacteria class survived with the ARGs in the effluent. Although investigations about the hosts of ARGs have been performed, studies about the relationships between ARGs and Gammaproteobacteria class were rarely found. Thus, further study about ARGs and their hosts, especially Gammaproteobacteria class, should be conducted to better understand the dissemination of ARGs in WWTPs and the aquatic environment.

The SmartChip analyses showed the presence of clinically significant ARGs in both, the influent and the effluent, found in opportunistic pathogenic MDR bacteria. The *vanC* gene found in the influent and effluent of the JN WWTP has also been detected at 33 sites in three main aquatic environments (Nam et al., 2013) and poultry farms (Seok et al., 2005) in South Korea. This may indicate that *vanC* is already spread out in the environment, animal farms, and even public health settings. In addition, the recently globally issued carbapenem-hydrolyzing β-lactamase gene, *bla*_*NDM*_, was also detected in the JN WWTP influent. We previously reported the presence of a variant of *bla*_*NDM*_, *bla*_*NDM*–5_, in an *E. coli* strain found in the influent at the same site (Shin et al., 2021). We also previously reported a *Klebsiella variicola* strain possessing *bla*_*NDM*–9_ (Di et al., 2017), which was isolated from the Gwangju River in South Korea. The ARGs in the surviving MDR bacteria, or in a free state in the environment, after the treatment process in WWTPs are likely to be prevalent in different bacteria present in different environments, thus making them widespread and persistent.

In addition, a previous study reported that the mobile resistome in the Han River aquatic environment would undergo a bloom of ARGs by anthropogenic forces from upstream regions (Lee et al., 2020). Based on this previous study and our data, resistance genes to aminoglycoside [variants of *aac (6’)* and *aph (3)*], β-lactam (variants of *bla*_*OXA*_ and *bla*_*GES*_), sulfonamide (*sul1* and *sul2*), and tetracycline (*tetC* and *tetG*) are considered the core ARGs in both, the Han River and the JN WWTP. This may indicate that these genes are already widely disseminated along the Han River and adjacent regions. Although the source of these ARGs is still unclear, the co-occurrence of these ARGs in both, the JN WWTP and the Han River, possibly proves the resistome of the Han River to be derived from the WWTP as a source of anthropogenic activity. The presence of such clinically relevant core ARGs in the influent and effluent of the JN WWTP, which were also observed in the Han River, possibly implies that they have been disseminated into aquatic environments.

In conclusion, the treatment process used at the JN WWTP led to a reduction in the abundance of ARB and ARGs but did not prove sufficient in eliminating all of them. Interestingly, diverse classes of bacteria showing multidrug resistance that were present in the influent were concentrated into mainly one class of Gammaproteobacteria consisting of *Citrobacter, Escherichia-Shigella*, and *Stenotrophomonas*, which retained multidrug resistance. Presently, we do not know the biological characteristics that enable this class to survive the treatment processes, and deciphering such characteristics requires further investigation. In addition, in the effluent, some clinically relevant core ARGs, such as *vanC*, *bla*_*OXA*_, and *bla*_*NDM*_, persisted through disinfection processes and, therefore, were thought to be discharged into the aquatic environment. Considering bacterial abilities for exchanging ARGs among them (conjugation) and transferring ARGs from environments into themselves (transformation), Gram-negative MDR bacteria such as *Citrobacter, Escherichia-Shigella*, and *Stenotrophomonas* and the core ARGs possessed by bacteria could be sources for the circulation and bloom of the antibiotic resistome in the JN WWTP and nearby environments.

## Data Availability Statement

The original contributions presented in this study are included in the article/Supplementary Material, further inquiries can be directed to the corresponding author.

## Author Contributions

HS: conceptualization, data curation, software, formal analysis, and writing—original draft. YK: investigation, methodology, and software. SR and S-HR: investigation. TU: project administration and validation. H-GH: writing—review and editing and funding acquisition. All authors contributed to the article and approved the submitted version.

## Conflict of Interest

The authors declare that the research was conducted in the absence of any commercial or financial relationships that could be construed as a potential conflict of interest.

## Publisher’s Note

All claims expressed in this article are solely those of the authors and do not necessarily represent those of their affiliated organizations, or those of the publisher, the editors and the reviewers. Any product that may be evaluated in this article, or claim that may be made by its manufacturer, is not guaranteed or endorsed by the publisher.
